# Approximations to inverse tangent function

**DOI:** 10.1186/s13660-018-1734-7

**Published:** 2018-06-20

**Authors:** Quan-Xi Qiao, Chao-Ping Chen

**Affiliations:** School of Mathematics and Informatics, Henan Polytechnic University, Jiaozuo, China

**Keywords:** 26D05, Inverse trigonometric function, Inequality, Approximation

## Abstract

In this paper, we present a sharp Shafer-type inequality for the inverse tangent function. Based on the Padé approximation method, we give approximations to the inverse tangent function. Based on the obtained result, we establish new bounds for arctan*x*.

## Introduction

In 1966, Shafer [1] posed, as a problem, the following inequality:

1.1 $$ \frac{3x}{1+2\sqrt{1+x^{2}}}< \arctan x, \quad x>0. $$

Three proofs of it were later given in [2]. Shafer’s inequality (1.1) was sharpened and generalized by Qi *et al.* in [3]. A survey and expository of some old and new inequalities associated with trigonometric functions can be found in [4]. Chen *et al.* [5] presented a new method to sharpen bounds of both sinc*x* and arcsin*x* functions, and the inequalities in exponential form as well.

For each $a>0$, Chen and Cheung [6] determined the largest number *b* and the smallest number *c* such that the inequalities

1.2 $$ \frac{b x}{1+a\sqrt{1+x^{2}}}\leq \arctan x\leq \frac{c x}{1+a\sqrt{1+x ^{2}}} $$

are valid for all $x\geq 0$. More precisely, these author proved that the largest number *b* and the smallest number *c* required by inequality (1.2) are

$$\begin{aligned} & \text{when } 0< a\leq \frac{\pi }{2},\quad b =\frac{\pi }{2}a,\qquad c=1+a; \\ & \text{when } \frac{\pi }{2}< a\leq \frac{2}{\pi -2},\quad b = \frac{4(a^{2}-1)}{a^{2}},\qquad c=1+a; \\ & \text{when } \frac{2}{\pi -2}< a< 2,\quad b =\frac{4(a^{2}-1)}{a^{2}},\qquad c= \frac{\pi }{2}a; \\ & \text{when } 2\leq a< \infty,\quad b =1+a, \qquad c=\frac{\pi }{2}a. \end{aligned}$$

In 1974, Shafer [7] indicated several elementary quadratic approximations of selected functions without proof. Subsequently, Shafer [8] established these results as analytic inequalities. For example, Shafer [8] proved that, for $x>0$,

1.3 $$ \frac{8x}{3+\sqrt{25+\frac{80}{3}x^{2}}}< \arctan x. $$

The inequality (1.3) can also be found in [9]. The inequality (1.3) is an improvement of the inequality (1.1).

Zhu [10] developed (1.3) to produce a symmetric double inequality. More precisely, the author proved that, for $x>0$,

1.4 $$ \frac{8x}{3+\sqrt{25+\frac{80}{3}x^{2}}}< \arctan x< \frac{8x}{3+\sqrt{25+\frac{256}{ \pi^{2}}x^{2}}}, $$

where the constants $80/3$ and $256/\pi^{2}$ are the best possible.

### Remark 1.1

For $x>0$, the following symmetric double inequality holds:

1.5 $$ \frac{8x}{3+\sqrt{25+\frac{80}{3}x^{2}}}< \arctan x< \frac{\frac{2 \sqrt{15}\pi }{3}x}{3+\sqrt{25+\frac{80}{3}x^{2}}}, $$

where the constants 8 and $\frac{2\sqrt{15}\pi }{3}$ are the best possible. We here point out that, for $x>0$, the upper bound in (1.4) is better than the upper bound in (1.5).

Based on the following power series expansion:

$$\arctan x \biggl( 3+\sqrt{25+\frac{80}{3}x^{2}} \biggr) =8x+ \frac{32}{4725}x^{7}-\frac{64}{4725}x^{9}+ \frac{25\text{,}376}{1\text{,}299\text{,}375}x^{11}- \cdots, $$

Sun and Chen [11] presented a new upper bound and proved that, for $x>0$,

1.6 $$ \arctan x< \frac{8x+\frac{32}{4725}x^{7}}{3+\sqrt{25+\frac{80}{3}x ^{2}}}. $$

Moreover, these authors pointed out that, for $0< x< x_{0}=1.4243\ldots $ , the upper bound in (1.6) is better than the upper bound in (1.4). In fact, we have the following approximation formulas near the origin:

$$\begin{aligned}& \arctan x-\frac{8x}{3+\sqrt{25+\frac{256}{\pi^{2}}x^{2}}}=O\bigl(x^{3}\bigr), \\& \arctan x-\frac{3x}{1+2\sqrt{1+x^{2}}}=O\bigl(x^{5}\bigr), \\& \arctan x-\frac{8x}{3+\sqrt{25+\frac{80}{3}x^{2}}}=O\bigl(x^{7}\bigr), \end{aligned}$$

and

$$\arctan x-\frac{8x+\frac{32}{4725}x^{7}}{3+\sqrt{25+\frac{80}{3}x ^{2}}}=O\bigl(x^{9}\bigr). $$

Nishizawa [12] proved that, for $x>0$,

1.7 $$ \frac{\pi^{2}x}{4+\sqrt{(\pi^{2}-4)^{2}+(2\pi x)^{2}}}< \arctan x< \frac{ \pi^{2}x}{4+\sqrt{32+(2\pi x)^{2}}}, $$

where the constants $(\pi^{2}-4)^{2}$ and 32 are the best possible.

Using the Maple software, we derive the following asymptotic formulas in the Appendix:

1.8 $$\begin{aligned}& \frac{\arctan x}{x}=\frac{\pi^{2}}{4+\sqrt{32+(2\pi x)^{2}}}-\frac{12- \pi^{2}}{3\pi^{2} x^{4}}+O \biggl( \frac{1}{x^{5}} \biggr), \end{aligned}$$

1.9 $$\begin{aligned}& \frac{\arctan x}{x} =\frac{3\pi^{2}}{24-\pi^{2}+\sqrt{432-24\pi^{2}+ \pi^{4}-12\pi (12-\pi^{2})x+(6\pi x)^{2}}} \\& \hphantom{\frac{\arctan x}{x} =} {} +\frac{\pi^{4}-72}{18\pi^{3}x^{5}}+O \biggl( \frac{1}{x^{6}} \biggr), \end{aligned}$$

and

1.10 $$\begin{aligned} x \biggl( \frac{\pi }{2}-\arctan x \biggr) = \frac{x^{2}+\frac{4}{15}}{x ^{2}+\frac{3}{5}}+O \biggl( \frac{1}{x^{6}} \biggr) \end{aligned}$$

as $x\to \infty $.

In this paper, motivated by (1.9), we establish a symmetric double inequality for arctan*x*. Based on the Padé approximation method, we develop the approximation formula (1.10) to produce a general result. More precisely, we determine the coefficients $a_{j}$ and $b_{j}$ ($1 \leq j\leq k$) such that

$$x \biggl( \frac{\pi }{2}-\arctan x \biggr) =\frac{x^{2k}+a_{1}x^{2(k-1)}+ \cdots +a_{k}}{x^{2k}+b_{1}x^{2(k-1)}+\cdots +b_{k}}+ O \biggl( \frac{1}{x ^{4k+2}} \biggr), \quad x\to \infty, $$

where $k\geq 1$ is any given integer. Based on the obtained result, we establish new bounds for arctan*x*.

Some computations in this paper were performed using Maple software.

## Lemma

It is well known that

2.1 $$\begin{aligned} \sum_{k=0}^{2n+1}(-1)^{k} \frac{x^{2k+1}}{(2k+1)!}< \sin x< \sum_{k=0} ^{2n}(-1)^{k}\frac{x^{2k+1}}{(2k+1)!} \end{aligned}$$

and

2.2 $$\begin{aligned} \sum_{k=0}^{2n+1}(-1)^{k} \frac{x^{2k}}{(2k)!}< \cos x< \sum_{k=0}^{2n}(-1)^{k} \frac{x ^{2k}}{(2k)!} \end{aligned}$$

for $x>0$ and $n\in \mathbb{N}_{0}:=\mathbb{N}\cup \{0\}$, where $\mathbb{N}$ denotes the set of positive integers.

The following lemma will be used in our present investigation.

### Lemma 2.1

*For* $0< u<\pi /2$,

2.3 $$\begin{aligned} &\cos u\sin^{2}u>u^{2}-\frac{5}{6}u^{4}+ \frac{91}{360}u^{6}- \frac{41}{1008}u^{8} \end{aligned}$$

*and*

2.4 $$\begin{aligned} \sin^{3} u>u^{3}-\frac{1}{2}u^{5}+ \frac{13}{120}u^{7}-\frac{41}{3024}u ^{9}. \end{aligned}$$

### Proof

We find that

2.5 $$\begin{aligned} \cos u\sin^{2}u =&\frac{1}{4} \bigl(\cos u-\cos (3u) \bigr) \\ =&u^{2}- \frac{5}{6}u^{4}+\frac{91}{360}u^{6}- \frac{41}{1008}u^{8}+\sum_{n=5} ^{\infty }(-1)^{n-1}w_{n}(u) \end{aligned}$$

and

2.6 $$\begin{aligned} \sin^{3} u =&\frac{1}{4} \bigl(3\sin u-\sin (3u) \bigr) \\ =&u^{3}-\frac{1}{2}u ^{5}+\frac{13}{120}u^{7}- \frac{41}{3024}u^{9}+\sum_{n=5}^{\infty }(-1)^{n-1}W _{n}(u), \end{aligned}$$

where

$$\begin{aligned} w_{n}(u)=\frac{9^{n}-1}{(2n)!}u^{2n} \quad \text{and}\quad W_{n}(u)=\frac{3(9^{n}-1)}{4 \cdot (2n+1)!}u^{2n+1}. \end{aligned}$$

Elementary calculations reveal that, for $0< u<\pi /2$ and $n\geq 5$,

$$\begin{aligned} \frac{w_{n+1}(u)}{w_{n}(u)} &= \frac{u^{2}(9^{n+1}-1)}{2(2n+1)(n+1)(9^{n}-1)}< \frac{(\pi /2)^{2}(9^{n+1}-1)}{2(2n+1)(n+1)(9^{n}-1)} \\ &< \frac{3\cdot 9^{n+1}}{2(2n+1)(n+1)(9^{n}-1)}= \frac{27}{2(2n+1)(n+1)} \biggl\{ 1+\frac{1}{9^{n}-1} \biggr\} \\ &\leq \frac{27}{2(2n+1)(n+1)} \biggl\{ 1+\frac{1}{9^{5}-1} \biggr\} = \frac{1\text{,}594\text{,}323}{118\text{,}096(2n+1)(n+1)}< 1 \end{aligned}$$

and

$$\begin{aligned} \frac{W_{n+1}(u)}{W_{n}(u)} &= \frac{u^{2}(9^{n+1}-1)}{2(2n+3)(n+1)(9^{n}-1)}< \frac{w_{n+1}(u)}{w _{n}(u)}< 1. \end{aligned}$$

Therefore, for fixed $u\in (0,\pi /2)$, the sequences $n\longmapsto w _{n}(u)$ and $n\longmapsto W_{n}(u)$ are both strictly decreasing for $n\geq 5$. From (2.5) and (2.6), we obtain the desired results (2.3) and (2.4). □

The proof of Theorem 3.1 makes use of the inequalities (2.1)–(2.4).

## Sharp Shafer-type inequality

Equation (1.9) motivated us to establish a symmetric double inequality for arctan*x*.

### Theorem 3.1

*For* $x>0$, *we have*

3.1 $$\begin{aligned} &\frac{3\pi^{2}x}{24-\pi^{2}+\sqrt{\alpha -12\pi (12-\pi^{2})x+36\pi ^{2}x^{2}}} \\ &\quad < \arctan x \\ &\quad < \frac{3\pi^{2}x}{24-\pi^{2}+\sqrt{\beta -12\pi (12-\pi^{2})x+36\pi ^{2}x^{2}}}, \end{aligned}$$

*with the best possible constants*

3.2 $$ \begin{aligned} &\alpha =432-24\pi^{2}+ \pi^{4}=292.538\ldots \quad \textit{and} \\ &\beta =576-192\pi^{2}+ 16\pi^{4}=239.581\ldots. \end{aligned} $$

### Proof

The inequality (3.1) can be written for $x>0$ as

3.3 $$\begin{aligned} \beta < \biggl( \frac{3\pi^{2} x^{2}}{\arctan x}-\bigl(24-\pi^{2} \bigr) \biggr) ^{2}+12 \pi \bigl(12-\pi^{2}\bigr)x-36 \pi^{2} x^{2}< \alpha. \end{aligned}$$

By the elementary change of variable $t = \arctan x$ ($x>0$), (3.3) becomes

3.4 $$\begin{aligned} \beta < \vartheta (t)< \alpha,\quad 0< t< \frac{\pi }{2}, \end{aligned}$$

where

$$\begin{aligned} \vartheta (t)={}& \biggl( \frac{3\pi^{2}\tan^{2} t}{t}-\bigl(24-\pi^{2}\bigr) \biggr) ^{2} \\ &{}+12\pi \bigl(12-\pi^{2}\bigr)\tan t-36 \pi^{2}\tan^{2}t. \end{aligned}$$

Elementary calculations reveal that

$$\begin{aligned} &\lim_{t\to 0^{+}}\vartheta (t)=576-192\pi^{2}+ 16 \pi^{4} \quad \text{and} \\ & \lim_{t\to \pi /2^{-}}\vartheta (t)=432-24\pi^{2}+\pi ^{4}. \end{aligned}$$

In order to prove (3.4), it suffices to show that $\vartheta (t)$ is strictly increasing for $0 < t < \pi /2$.

Differentiation yields

$$\begin{aligned} t^{3}\cos^{3}t\vartheta '(t) =&\bigl(24\pi t- \pi^{3} t\bigr)\sin t\cos^{2}t+\bigl(3\pi ^{3} t-12 \pi t^{3}\bigr)\sin t \\ &{} - \bigl(3\pi^{3}+\bigl(24\pi -\pi^{3}\bigr) t^{2}-\bigl(24-2\pi^{2}\bigr) t^{3} \bigr)\cos t+3 \pi^{3}\cos^{3} t \\ =:&\lambda (t). \end{aligned}$$

We now consider two cases to prove $\lambda (t)>0$ for $0< t<\pi /2$.

*Case* 1: $0< t\leq 0.6$.

Using (2.1) and (2.2), we have, for $0< t\leq 0.6$,

$$\begin{aligned} \lambda (t) =& \biggl( 6\pi -\frac{1}{4}\pi^{3} \biggr) t\sin (3t)+ \frac{3}{4}\pi^{3}\cos (3t)+ \biggl\{ \biggl( 6\pi + \frac{11}{4}\pi^{3} \biggr) t-12 \pi t^{3} \biggr\} \sin t \\ &{} - \biggl( \frac{3}{4}\pi^{3}-\bigl(\pi^{3}-24 \pi\bigr)t^{2}-\bigl(24-2\pi^{2}\bigr)t ^{3} \biggr) \cos t \\ >& \biggl( 6\pi -\frac{1}{4}\pi^{3} \biggr) t \biggl( 3t- \frac{9}{2}t^{3}+ \frac{81}{40}t^{5}- \frac{243}{560}t^{7} \biggr) \\ &{}+\frac{3}{4}\pi^{3} \biggl( 1-\frac{9}{2}t^{2}+\frac{27}{8}t^{4}- \frac{81}{80}t^{6} \biggr) \\ &{} + \biggl\{ \biggl( 6\pi +\frac{11}{4}\pi^{3} \biggr) t-12 \pi t^{3} \biggr\} \biggl( t-\frac{1}{6}t^{3} \biggr) \\ &{} - \biggl( \frac{3}{4}\pi^{3}-\bigl(\pi^{3}-24\pi \bigr)t^{2}-\bigl(24-2\pi^{2}\bigr)t ^{3} \biggr) \biggl( 1-\frac{1}{2}t^{2}+\frac{1}{24}t^{4} \biggr) \\ =&t^{3} \biggl\{ 24-2\pi^{2}- \biggl( 28\pi - \frac{8}{3}\pi^{3} \biggr) t- \bigl( 12-\pi^{2} \bigr) t^{2} \biggr\} \\ &{} +t^{6} \biggl\{ \frac{263}{20}\pi -\frac{235}{192} \pi^{3}+ \biggl( 1- \frac{1}{12}\pi^{2} \biggr) t- \biggl( \frac{729}{280}\pi - \frac{243}{2240}\pi^{3} \biggr) t^{2} \biggr\} . \end{aligned}$$

Each function in curly braces is positive for $t\in (0,0.6]$. Thus, $\lambda (t)>0$ for $t\in (0,0.6]$.

*Case* 2: $0.6< t<\pi /2$.

We now prove $\lambda (t)>0$ for $0.6< t<\pi /2$. Replacing *t* by $\frac{\pi }{2}-u$ leads to an equivalent inequality:

$$\begin{aligned} \mu (u)>0,\quad 0< u< \frac{\pi }{2}-0.6, \end{aligned}$$

where

$$\begin{aligned} \mu (u) ={}&\bigl(24\pi -\pi^{3} \bigr) \biggl( \frac{\pi }{2}-u \biggr) \cos u\sin^{2}u+ \biggl\{ 3\pi^{3} \biggl( \frac{\pi }{2}-u \biggr) -12\pi \biggl( \frac{ \pi }{2}-u \biggr) ^{3} \biggr\} \cos u \\ &{} - \biggl\{ 3\pi^{3}+\bigl(24\pi -\pi^{3}\bigr) \biggl( \frac{\pi }{2}-u \biggr) ^{2}-\bigl(24-2\pi^{2}\bigr) \biggl( \frac{\pi }{2}-u \biggr) ^{3} \biggr\} \sin u+3 \pi^{3}\sin^{3} u. \end{aligned}$$

Using (2.1)–(2.4), we have, for $0< u<\frac{\pi }{2}-0.6$,

$$\begin{aligned} \mu (u) >&\bigl(24\pi -\pi^{3} \bigr) \biggl( \frac{\pi }{2}-u \biggr) \biggl( u^{2}- \frac{5}{6}u^{4}+ \frac{91}{360}u^{6}-\frac{41}{1008}u^{8} \biggr) \\ &{} + \biggl\{ 3\pi^{3} \biggl( \frac{\pi }{2}-u \biggr) -12\pi \biggl( \frac{ \pi }{2}-u \biggr) ^{3} \biggr\} \biggl( 1- \frac{1}{2}u^{2}+\frac{1}{24}u ^{4}- \frac{1}{720}u^{6} \biggr) \\ &{} - \biggl\{ 3\pi^{3}+\bigl(24\pi -\pi^{3}\bigr) \biggl( \frac{\pi }{2}-u \biggr) ^{2}-\bigl(24-2\pi^{2}\bigr) \biggl( \frac{\pi }{2}-u \biggr) ^{3} \biggr\} \biggl( u- \frac{1}{6}u^{3}+\frac{1}{120}u^{5} \biggr) \\ &{} +3\pi^{3} \biggl( u^{3}-\frac{1}{2}u^{5}+ \frac{13}{120}u^{7}- \frac{41}{3024}u^{9} \biggr) \\ =&u^{4} \biggl\{ \frac{1}{3}\pi^{4}-24+ \biggl( 12\pi -\frac{9}{5}\pi^{3} \biggr) u+ \biggl( 2\pi^{2}- \frac{11}{90}\pi^{4}+4 \biggr) u^{2} \\ &{}+ \biggl( - \frac{82}{15}\pi +\frac{199}{360}\pi^{3} \biggr) u^{3} \\ &{} + \biggl( -\frac{1}{5}-\frac{25}{56}\pi^{2}+ \frac{41}{2016}\pi^{4} \biggr) u ^{4}+ \biggl( \frac{403}{420}\pi -\frac{41}{504}\pi^{3} \biggr) u^{5} \biggr\} >0. \end{aligned}$$

We then obtain $\lambda (t)>0$ and $\vartheta '(t)>0$ for all $0< t<\pi /2$. Hence, $\vartheta (t)$ is strictly increasing for $0 < t < \pi /2$. The proof is complete. □

From (1.7) and (3.1), we obtain the following approximation formulas:

3.5 $$ \frac{\arctan n}{n} \approx \frac{\pi^{2}}{4+\sqrt{32+(2\pi n)^{2}}}=:a_{n} $$

and

3.6 $$\begin{aligned} \frac{\arctan n}{n}\approx \frac{3\pi^{2}}{24-\pi^{2}+\sqrt{432-24 \pi^{2}+\pi^{4}-12\pi (12-\pi^{2})n+(6\pi n)^{2}}}=:b_{n}, \end{aligned}$$

as $n\to \infty $.

The following numerical computations (see Table 1) would show that, for $n\in \mathbb{N}$, Eq. (3.6) is sharper than Eq. (3.5).

**Table 1:** Comparison between approximation formulas (3.5) and (3.6).

*n*	$a_{n}-\frac{\arctan n}{n}$	$\frac{\arctan n}{n}-b_{n}$
1	7.055 × 10^−3^	5.259 × 10^−3^
10	5.95 × 10^−6^	3.939 × 10^−7^
100	7.066 × 10^−10^	4.492 × 10^−12^
1000	7.182 × 10^−14^	4.546 × 10^−17^
10,000	7.193 × 10^−18^	4.552 × 10^−22^

In fact, we have, as $n\to \infty $,

$$\frac{\arctan n}{n} = a_{n}+O \biggl( \frac{1}{n^{4}} \biggr) \quad \text{and}\quad \frac{\arctan n}{n}= b_{n}+O \biggl( \frac{1}{n^{5}} \biggr). $$

## Approximations to arctan*x*

For later use, we introduce the Padé approximant (see [13–16]). Let *f* be a formal power series,

4.1 $$\begin{aligned} f(t)=c_{0}+c_{1}t+c_{2}t^{2}+ \cdots. \end{aligned}$$

The Padé approximation of order $(p, q)$ of the function *f* is the rational function, denoted by

4.2 $$\begin{aligned}{} [p/q]_{f}(t)=\frac{\sum_{j=0}^{p}a_{j}t^{j}}{1+\sum_{j=1}^{q}b_{j}t ^{j}}, \end{aligned}$$

where $p\geq 0$ and $q\geq 1$ are two given integers, the coefficients $a_{j}$ and $b_{j}$ are given by (see [13–15])

4.3 $$\begin{aligned} \textstyle\begin{cases} a_{0}=c_{0}, \\ a_{1}=c_{0}b_{1}+c_{1}, \\ a_{2}=c_{0}b_{2}+c_{1}b_{1}+c_{2}, \\ \vdots \\ a_{p} = c_{0}b_{p}+\cdots + c_{p-1}b_{1} + c_{p}, \\ 0 = c_{p+1} + c_{p}b_{1} + \cdots + c_{p-q+1}b_{q}, \\ \vdots & \\ 0 = c_{p+q} + c_{p+q-1}b_{1} + \cdots + c_{p}b_{q}, \end{cases}\displaystyle \end{aligned}$$

and the following holds:

4.4 $$\begin{aligned}{} [p/q]_{f}(t)- f (t) = O\bigl(t^{p+q+1} \bigr). \end{aligned}$$

Thus, the first $p + q + 1$ coefficients of the series expansion of $[p/q]_{f}$ are identical to those of *f*.

From the expansion (see [17, p. 81])

$$\begin{aligned} \arctan x=\frac{\pi }{2}+\sum_{j=1}^{\infty } \frac{(-1)^{j}}{(2j-1)x ^{2j-1}},\quad \vert x\vert >1, \end{aligned}$$

we obtain

4.5 $$\begin{aligned} x \biggl( \frac{\pi }{2}-\arctan x \biggr) =\sum _{j=0}^{\infty }\frac{c _{j}}{x^{2j}}=1-\frac{1}{3x^{2}}+ \frac{1}{5x^{4}}-\frac{1}{7x^{6}}+ \cdots, \end{aligned}$$

where

4.6 $$\begin{aligned} c_{j}=\frac{(-1)^{j}}{2j+1}\quad \text{for } j\geq 0. \end{aligned}$$

Let

4.7 $$\begin{aligned} f(t)=\sum_{j=0}^{\infty } \frac{c_{j}}{t^{j}}, \end{aligned}$$

with the coefficients $c_{j}$ given in (4.6). Then we have

4.8 $$\begin{aligned} f\bigl(x^{2}\bigr)=\sum_{j=0}^{\infty } \frac{c_{j}}{x^{2j}}=x \biggl( \frac{\pi }{2}- \arctan x \biggr). \end{aligned}$$

In what follows, the function *f* is given in (4.7).

Based on the Padé approximation method, we now give a derivation of Eq. (1.10). To this end, we consider

$$\begin{aligned}{} [1/1]_{f}(t)=\frac{\sum_{j=0}^{1}a_{j}t^{-j}}{1+\sum_{j=1}^{1}b_{j}t ^{-j}}. \end{aligned}$$

Noting that

4.9 $$\begin{aligned} c_{0}=1, \qquad c_{1}=-\frac{1}{3}, \qquad c_{2}=\frac{1}{5}, \end{aligned}$$

holds, we have, by (4.3),

$$\begin{aligned} \textstyle\begin{cases} a_{0}=1, \\ a_{1}=b_{1}-\frac{1}{3}, \\ 0 =\frac{1}{5} -\frac{1}{3}b_{1}, \end{cases}\displaystyle \end{aligned}$$

that is,

$$\begin{aligned} a_{0}=1,\qquad a_{1}=\frac{4}{15}, \qquad b_{1}=\frac{3}{5}. \end{aligned}$$

We thus obtain

4.10 $$ [1/1]_{f}(t)= \frac{1+\frac{4}{15t}}{1+\frac{3}{5t}}= \frac{15t+4}{3(5t+3)}, $$

and we have, by (4.4),

4.11 $$ f(t)=\frac{15t+4}{3(5t+3)}+O \biggl( \frac{1}{t^{3}} \biggr), \quad t\to \infty. $$

Replacing *t* by $x^{2}$ in (4.11) yields (1.10).

From the Padé approximation method and the expansion (4.7), we now present a general result.

### Theorem 4.1

*The Padé approximation of order* $(p, q)$ *of the function* $f(t)=\sum_{j=0}^{\infty }\frac{c_{j}}{t^{j}}$ (*at the point* $t=\infty $) *is the following rational function*:

4.12 $$\begin{aligned}{} [p/q]_{f}(t)&=\frac{1+\sum_{j=1}^{p}a_{j}t^{-j}}{1+\sum_{j=1}^{q}b_{j}t ^{-j}} \\ &=t^{q-p} \biggl( \frac{t^{p}+a_{1}t^{p-1}+\cdots +a_{p}}{t^{q}+b _{1}t^{q-1}+\cdots +b_{q}} \biggr), \end{aligned}$$

*where* $p\geq 1$ *and* $q\geq 1$ *are any given integers*, *the coefficients* $a_{j}$ *and* $b_{j}$ *are given by*

4.13 $$\begin{aligned} \textstyle\begin{cases} a_{1}=b_{1}+c_{1}, \\ a_{2}=b_{2}+c_{1}b_{1}+c_{2}, \\ \vdots \\ a_{p} = b_{p}+\cdots + c_{p-1}b_{1} + c_{p}, \\ 0 = c_{p+1} + c_{p}b_{1} + \cdots + c_{p-q+1}b_{q}, \\ \vdots & \\ 0 = c_{p+q} + c_{p+q-1}b_{1} + \cdots + c_{p}b_{q}, \end{cases}\displaystyle \end{aligned}$$

*and* $c_{j}$ *is given in* (4.6), *and the following holds*:

4.14 $$\begin{aligned} f (t) -[p/q]_{f}(t) = O \biggl( \frac{1}{t^{p+q+1}} \biggr),\quad t\to \infty. \end{aligned}$$

*In particular*, *replacing* *t* *by* $x^{2}$ *in* (4.14) *yields*

4.15 $$\begin{aligned} &x \biggl( \frac{\pi }{2}-\arctan x \biggr) \\ &\quad =x^{2(q-p)} \biggl( \frac{x^{2p}+a _{1}x^{2(p-1)}+\cdots +a_{p}}{x^{2q}+b_{1}x^{2(q-1)}+\cdots +b_{q}} \biggr) + O \biggl( \frac{1}{x^{2(p+q+1)}} \biggr), \quad x\to \infty, \end{aligned}$$

*with the coefficients* $a_{j}$ *and* $b_{j}$ *given by* (4.13).

Setting $(p, q)=(k, k)$ in (4.15), we obtain the following corollary.

### Corollary 4.1

*As* $x\to \infty $,

4.16 $$\begin{aligned} x \biggl( \frac{\pi }{2}-\arctan x \biggr) = \frac{x^{2k}+a_{1}x^{2(k-1)}+ \cdots +a_{k}}{x^{2k}+b_{1}x^{2(k-1)}+\cdots +b_{k}}+ O \biggl( \frac{1}{x ^{4k+2}} \biggr), \end{aligned}$$

*where* $k\geq 1$ *is any given integer*, *the coefficients* $a_{j}$ *and* $b_{j}$ ($1\leq j\leq k$) *are given by*

4.17 $$\begin{aligned} \textstyle\begin{cases} a_{1}=b_{1}+c_{1}, \\ a_{2}=b_{2}+c_{1}b_{1}+c_{2}, \\ \vdots \\ a_{k} = b_{k}+\cdots + c_{k-1}b_{1} + c_{k}, \\ 0 = c_{k+1} + c_{k}b_{1} + \cdots + c_{1}b_{k}, \\ \vdots & \\ 0 = c_{2k} + c_{2k-1}b_{1} + \cdots + c_{k}b_{k}, \end{cases}\displaystyle \end{aligned}$$

*and* $c_{j}$ *is given in* (4.6).

Setting $k=2$ in (4.16) yields, as $x\to \infty $,

4.18 $$\begin{aligned} x \biggl( \frac{\pi }{2}-\arctan x \biggr) = \frac{945x^{4}+735x^{2}+64}{15(63x ^{4}+70x^{2}+15)}+O \biggl( \frac{1}{x^{10}} \biggr), \end{aligned}$$

which gives

$$\begin{aligned} \arctan x=\frac{\pi }{2}-\frac{945x^{4}+735x^{2}+64}{15x(63x^{4}+70x ^{2}+15)}+ O \biggl( \frac{1}{x^{11}} \biggr). \end{aligned}$$

Using the Maple software, we find, as $x\to \infty $,

4.19 $$\begin{aligned} \arctan x={}&\frac{\pi }{2}-\frac{945x^{4}+735x^{2}+64}{15x(63x^{4}+70x ^{2}+15)}+ \frac{64}{43\text{,}659x^{11}} \\ &{}-\frac{1856}{464\text{,}373x^{13}}+ O \biggl( \frac{1}{x ^{15}} \biggr). \end{aligned}$$

Equation (4.19) motivated us to establish new bounds for arctan*x*.

### Theorem 4.2

*For* $x>0$, *we have*

4.20 $$\begin{aligned} &\frac{\pi }{2}-\frac{945x^{4}+735x^{2}+64}{15x(63x^{4}+70x^{2}+15)}+\frac{64}{43\text{,}659x ^{11}}- \frac{1856}{464\text{,}373x^{13}} \\ &\quad < \arctan x< \frac{\pi }{2}-\frac{945x^{4}+735x^{2}+64}{15x(63x^{4}+70x ^{2}+15)}+\frac{64}{43\text{,}659x^{11}}. \end{aligned}$$

### Proof

For $x>0$, let

$$\begin{aligned} I(x)=\arctan x- \biggl( \frac{\pi }{2}-\frac{945x^{4}+735x^{2}+64}{15x(63x ^{4}+70x^{2}+15)}+ \frac{64}{43\text{,}659x^{11}}-\frac{1856}{464\text{,}373x^{13}} \biggr) \end{aligned}$$

and

$$\begin{aligned} J(x)=\arctan x- \biggl( \frac{\pi }{2}-\frac{945x^{4}+735x^{2}+64}{15x(63x ^{4}+70x^{2}+15)}+ \frac{64}{43\text{,}659x^{11}} \biggr). \end{aligned}$$

Differentiation yields

$$\begin{aligned} I'(x)=-\frac{64(230\text{,}391x^{8}+372\text{,}680x^{6}+236\text{,}885x^{4}+65\text{,}400x^{2}+6525)}{35\text{,}721x ^{14}(1+x^{2})(63x^{4}+70x^{2}+15)^{2}}< 0 \end{aligned}$$

and

$$\begin{aligned} J'(x)=\frac{64(12\text{,}789x^{8}+15\text{,}610x^{6}+8890x^{4}+2325x^{2}+225)}{3969x ^{12}(1+x^{2})(63x^{4}+70x^{2}+15)^{2}}>0. \end{aligned}$$

Hence, $I(x)$ is strictly decreasing and $J(x)$ is strictly increasing for $x>0$, and we have

$$\begin{aligned} I(x)>\lim_{t\to \infty }I(t)=0 \quad \text{and}\quad J(x)< \lim _{t\to \infty }J(t)=0 \quad \text{for } x>0. \end{aligned}$$

The proof is complete. □

### Remark 4.1

We point out that, for $x>1.0213\ldots $ , the lower bound in (4.20) is better than the one in (1.7). For $x>0.854439\ldots $ , the upper bound in (4.20) is better than the one in (1.7). For $x>0.947273\ldots $ , the lower bound in (4.20) is better than the one in (3.1). For $x>0.792793\ldots $ , the upper bound in (4.20) is better than the one in (3.1).

## Conclusions

In this paper, we establish a symmetric double inequality for arctan*x* (Theorem 3.1). We determine the coefficients $a_{j}$ and $b_{j}$ ($1\leq j\leq k$) such that

$$x \biggl( \frac{\pi }{2}-\arctan x \biggr) =\frac{x^{2k}+a_{1}x^{2(k-1)}+ \cdots +a_{k}}{x^{2k}+b_{1}x^{2(k-1)}+\cdots +b_{k}}+ O \biggl( \frac{1}{x ^{4k+2}} \biggr), \quad x\to \infty, $$

where $k\geq 1$ is any given integer (see Corollary 4.1). Based on the obtained result, we establish new bounds for arctan*x* (Theorem 4.2).

## Appendix: A derivation of (1.8), (1.9), and (1.10)

Define the function $F(x)$ by

$$F(x)=\frac{\arctan x}{x}-\frac{1}{a+\sqrt{b+c x^{2}}}. $$

We are interested in finding the values of the parameters *a*, *b*, and *c* such that $F(x)$ converges as fast as possible to zero, as $x\to \infty $. This provides the best approximations of the form

$$\begin{aligned} \frac{\arctan x}{x}\approx \frac{1}{a+\sqrt{b+c x^{2}}},\quad x\to \infty. \end{aligned}$$

Using the Maple software, we find, as $x\to \infty $,

$$\begin{aligned} F(x) ={}&\frac{\pi \sqrt{c}-2}{2\sqrt{c}x}+\frac{a-c}{cx^{2}}+\frac{b-2a ^{2}}{2c^{3/2}x^{3}} \\ &{}+ \frac{3a^{3}+c^{2}-3ab}{3c^{2} x^{4}}+O \biggl( \frac{1}{x ^{5}} \biggr). \end{aligned}$$

The three parameters *a*, *b*, and *c*, which produce the fastest convergence of the function $F(x)$, are given by

$$\textstyle\begin{cases} \pi \sqrt{c}-2=0, \\ a-c=0, \\ b-2a^{2}=0, \end{cases} $$

namely, if

$$\begin{aligned} a=\frac{4}{\pi^{2}},\qquad b=\frac{32}{\pi^{4}}, \qquad c= \frac{4}{\pi ^{2}}. \end{aligned}$$

We then obtain, as $x\to \infty $,

$$\begin{aligned} \frac{\arctan x}{x} &=\frac{1}{\frac{4}{\pi^{2}}+\sqrt{\frac{32}{\pi ^{4}}+\frac{4}{\pi^{2}} x^{2}}}-\frac{12-\pi^{2}}{3\pi^{2} x^{4}}+O \biggl( \frac{1}{x^{5}} \biggr) \\ &=\frac{\pi^{2}}{4+\sqrt{32+(2\pi x)^{2}}}-\frac{12-\pi^{2}}{3\pi ^{2} x^{4}}+O \biggl( \frac{1}{x^{5}} \biggr). \end{aligned}$$

Define the function $G(x)$ by

$$G(x)=\frac{\arctan x}{x}-\frac{1}{p+\sqrt{q+r x+s x^{2}}}. $$

Using the Maple software, we find, as $x\to \infty $,

$$\begin{aligned} G(x) =&\frac{\pi \sqrt{s}-2}{2\sqrt{s}x}+\frac{r-2s^{3/2}+2p \sqrt{s}}{2s^{3/2}x^{2}}+\frac{4qs-3r^{2}-8sp^{2}-8\sqrt{s}pr}{8s ^{5/2}x^{3}} \\ &{} +\frac{-48s^{3/2}pq+48\sqrt{s}pr^{2}-36rqs+15r^{3}+48s^{3/2}p ^{3}+72sp^{2}r+16s^{7/2}}{48s^{7/2}x^{4}} \\ &{} +\bigl(120sqr^{2}-128\sqrt{s}pr^{3}-128s^{2}p^{4}-240sp ^{2}r^{2}+256rs^{3/2}pq+192s^{2}p^{2}q \\ &{}-256s^{3/2}p^{3}r-35r^{4}-48q ^{2}s^{2}\bigr)/\bigl(128s^{9/2}x^{5} \bigr) \\ &{} +O \biggl( \frac{1}{x^{6}} \biggr). \end{aligned}$$

For

$$\begin{aligned} p=\frac{24-\pi^{2}}{3\pi^{2}},\qquad q=\frac{432-24\pi^{2}+\pi^{4}}{9 \pi^{4}}, \qquad r=- \frac{4(12-\pi^{2})}{3\pi^{3}},\qquad s=\frac{4}{\pi ^{2}}, \end{aligned}$$

we obtain, as $x\to \infty $,

$$\begin{aligned} \frac{\arctan x}{x} =&\frac{3\pi^{2}}{24-\pi^{2}+\sqrt{432-24\pi^{2}+ \pi^{4}-12\pi (12-\pi^{2})x+(6\pi x)^{2}}} \\ &{} +\frac{\pi^{4}-72}{18\pi^{3}x^{5}}+O \biggl( \frac{1}{x^{6}} \biggr). \end{aligned}$$

Define the function $H(x)$ by

$$H(x)=x \biggl( \frac{\pi }{2}-\arctan x \biggr) -\frac{x^{2}+a_{1}x+a_{2}}{x ^{2}+b_{1}x+b_{2}}. $$

Using the Maple software, we find, as $x\to \infty $,

$$\begin{aligned} H(x) =&\frac{b_{1}-a_{1}}{x}-\frac{3a_{2}-3b_{2}-3a_{1}b_{1}+3b_{1} ^{2}+1}{3x^{2}}+\frac{a_{1}b_{2}-2b_{1}b_{2}+a_{2}b_{1}-a_{1}b_{1} ^{2}+b_{1}^{3}}{x^{3}} \\ &{} -\frac{-1-5a_{2}b_{2}+5b_{2}^{2}+10a_{1}b_{1}b_{2}-15b_{1} ^{2}b_{2}+5a_{2}b_{1}^{2}-5a_{1}b_{1}^{3}+5b_{1}^{4}}{5x^{4}} \\ &{} +\frac{-a_{1}b_{2}^{2}+3b_{1}b_{2}^{2}-2a_{2}b_{1}b_{2}+3a _{1}b_{1}^{2}b_{2}-4b_{1}^{3}b_{2}+a_{2}b_{1}^{3}-a_{1}b_{1}^{4}+b _{1}^{5}}{x^{5}}+O \biggl( \frac{1}{x^{6}} \biggr). \end{aligned}$$

For

$$\begin{aligned} a_{1}=0, \qquad b_{1}=0, \qquad a_{2}= \frac{4}{15}, \qquad b_{2}= \frac{3}{5}, \end{aligned}$$

we obtain, as $x\to \infty $,

$$\begin{aligned} x \biggl( \frac{\pi }{2}-\arctan x \biggr) =\frac{x^{2}+\frac{4}{15}}{x ^{2}+\frac{3}{5}}+O \biggl( \frac{1}{x^{6}} \biggr). \end{aligned}$$
